# Use of Oral Gonadotropin Releasing Hormone Antagonist Combinations for Preoperative Management of Refractory Leiomyoma-Related Heavy Menstrual Bleeding

**DOI:** 10.1089/whr.2025.0013

**Published:** 2025-03-26

**Authors:** Danielle A. Aase, Elizabeth A. Stewart

**Affiliations:** Division of Reproductive Endocrinology and Infertility, Department of Obstetrics and Gynecology, Mayo Clinic and Mayo Clinic College of Medicine and Science, Rochester, Minnesota, USA.

**Keywords:** gynecology, GnRH antagonist, leiomyoma, myomectomy

## Abstract

Uterine leiomyomas are noncancerous smooth muscle neoplasms that occur in up to 70% of women and the single most common cause of hysterectomy. There is a variety of treatments available for uterine leiomyomas, including medications, uterine artery embolization, myomectomy, and hysterectomy but surgical treatments predominate. Medical optimization of anemia is often required prior to surgical treatment secondary to heavy menstrual bleeding and resultant iron deficiency anemia from chronic blood loss. While iron and multivitamins are critical to increase hemoglobin, pharmacologic therapy to suppress menstrual bleeding is often needed. First line therapy is typically a trial of combined oral contraceptive pills (OCPs); however, this may not be effective in all patients. Parenteral administration of long-acting GnRH agonists is often used as a second line treatment; however, these agents can lead to an initial “flare” effect precipitating a heavy bleed during the agonist phase which can be problematic in such patients. An alternative course of action is the use of oral GnRH antagonist combination therapy (OGAC) with estradiol and norethindrone, an option approved in both the United States and the European Union for long-term treatment of leiomyoma-related heavy menstrual bleeding. To demonstrate the clinical utility of OGAC therapy in the preoperative setting, we report a successful patient outcome and review relevant literature.

Uterine leiomyomas are non-cancerous smooth muscle neoplasms that occur in up to 70–80% of persons with a uterus and the leading indication for hysterectomy. Of persons with uterine leiomyomas, approximately 50% have symptoms including prolonged and heavy menstrual bleeding or bulk-related symptoms such as pelvic pressure, constipation, or urinary frequency.^1^ The most common symptoms associated with uterine leiomyomas are heavy menstrual bleeding and pelvic pressure. Anemia secondary to heavy menstrual bleeding is frequent and often severe.^2^ There is a range of treatments available for uterine leiomyomas, including medications, uterine artery embolization, myomectomy, and hysterectomy but surgical treatments predominate.

Medical optimization, specifically treatment of anemia secondary to heavy menstrual bleeding, is often needed prior to surgical treatment. First line therapy is typically a trial of combined oral contraceptive pills (OCPs) however this may not be effective in all patients. GnRH agonists are often used as a second line choice; however, these agents can lead to an initial “flare” effect due to its initial gonadotropin release, precipitating a heavy withdrawal bleed and significant side effects.^3^ An alternative course of action is the use of oral GnRH antagonist combination therapy (OGAC) with estradiol and norethindrone, an option approved in both the United States and the European Union (EU) for treatment of leiomyoma-related heavy menstrual bleeding. GnRH antagonists competitively bind to the GnRH receptor and quickly deactivate it; in this way, they do not stimulate GnRH release and are not associated with a gonadotropin flare.^3^ To demonstrate the clinical utility of OGAC therapy in the preoperative setting, we report a successful patient outcome.

A 35-year-old G0 with a 5-year history of primary infertility and heavy menstrual bleeding presented for treatment of uterine leiomyomas. A pelvic MRI showed innumerable uterine leiomyomas with an overall uterine size of 20 × 10 × 16 cm without submucosal fibroids. She desired treatment with myomectomy. Her complete blood count returned with a hemoglobin of 5.9 g/dL, hematocrit of 23.9%, and an MCV of 61.8 consistent with severe iron deficiency anemia. Combined OCPs with oral iron supplements and a multivitamin were initiated to stabilize her hematological status. On follow-up 8 weeks later, her hemoglobin had risen to 8.0 g/dL with a hematocrit of 28.1%. While moderately successful in improving laboratory values, the patient perceived that the combined OCPs led to heavier menstrual bleeding and the oral iron caused significant GI distress. Therefore, relugolix combination therapy and intravenous iron infusions were initiated to medically optimize the patient prior to myomectomy. After four months of therapy, her menstrual bleeding decreased and her anemia improved with a hemoglobin of 12.8 g/dL and hematocrit of 41.1%. She underwent an uncomplicated abdominal myomectomy with an estimated blood loss of 200 mL. During the procedure, over 39 leiomyomas were removed with multiple intracavitary submucosal leiomyomas identified. Postoperatively, she was noted to have vaginal bleeding secondary to blood collecting within the endometrial cavity, which improved by postoperative day one. On postoperative day one, her hemoglobin was 8.8 g/dL and hematocrit 27.0%. She received one unit packed red blood cells and one dose of IV iron. She met postoperative milestones and discharged on postoperative day two. Relugolix was discontinued on postoperative day one. At her 6-week postoperative visit, she was doing well without issues and planned to attempt pregnancy three months after surgery.

There are multiple frequently used options to decrease menstrual bleeding and improve hematological status prior to myomectomy, including combined OCPs, progestins, or GnRH agonists.^3^ Leuprolide acetate, a GnRH agonist, is a common choice in this setting as it has not only been shown to increase hemoglobin levels prior to surgery, but also to decrease uterine size and leiomyoma volume.^4^ However, the initial flare from the agonist phase can cause an initial heavy episode of bleeding resulting in a median of 21 days to amenorrhea before downregulation is achieved, which is problematic in patients such as this who have profound preexisting anemia.^5^ In contrast, GnRH antagonists do not have this agonist phase and thus a much quicker suppression of bleeding.^3^ While selective progesterone receptor modulators (SPRMs) are widely used because of their more rapid induction of amenorrhea, they are not available in the United States for leiomyoma treatment.^4^

GnRH antagonist combination therapies are highly effective treatments for heavy menstrual bleeding with elagolix and relugolix combinations approved in the United States and relugolix and linzagolix combinations approved in the EU.^4,6,7^ The GnRH antagonists competitively bind to pituitary GnRH receptors, which prevents the binding of endogenous GnRH leading to rapid decrease in gonadotropin levels and subsequent decreased production of ovarian estradiol and progesterone. Combination therapy utilizes 1 mg of estradiol and 0.5 mg of norethindrone acetate to approximate the hormonal milieu of the early follicular phase, mitigating hypoestrogenic side effects without decreasing efficacy and leading to modest uterine volume reduction of approximately 10%. A majority (70–80%) of patients on combination therapy experience at least a 50% reduction in menstrual blood loss from baseline, with a mean reduction of blood loss of approximately 80%.^8^ Utilizing a GnRH antagonist alone leads to greater volume reduction, approximately 40%, but is not an approved therapy in the United States.^6^

In summary, it is reasonable to use a GnRH agonist to optimize a patient’s hematological status prior to myomectomy, especially if a reduction in leiomyoma volume is a key surgical goal. However, in the presence of anemia, where a steroidal flare can further compromise hematological status, or avoiding parenteral administration of medications is the goal, the use of an oral GnRH antagonist combination therapy is a reasonable alternative to GnRH agonists for preoperative optimization of hematological status prior to surgical therapy.

## Abbreviations Used

EU: European Union
OCPs: Oral contraceptive pills
OGAC: Oral GnRH antagonist combination therapy
SPRMs: Selective progesterone receptor modulators
